# Antibodies and superantibodies in patients with chronic rhinosinusitis with nasal polyps

**DOI:** 10.1016/j.jaci.2016.06.066

**Published:** 2017-04

**Authors:** Jiun-Bo Chen, Louisa K. James, Anna M. Davies, Yu-Chang Bryan Wu, Joanne Rimmer, Valerie J. Lund, Jou-Han Chen, James M. McDonnell, Yih-Chih Chan, George H. Hutchins, Tse Wen Chang, Brian J. Sutton, Harsha H. Kariyawasam, Hannah J. Gould

**Affiliations:** a Randall Division of Cell and Molecular Biophysics, King's College London, London, United Kingdom; b Genomics Research Center, Academia Sinica, Taipei, Taiwan; c MRC & Asthma UK Centre for Allergic Mechanisms of Asthma, King's College London, Guy's Campus, London, United Kingdom; d Allergy and Rhinology, Royal National Throat Nose Ear Hospital, London, United Kingdom

**Keywords:** Chronic rhinosinusitis with nasal polyps, aspirin-exacerbated respiratory disease, *Staphylococcus aureus* enterotoxin, superantigen, superantibody, basophil, 7-AAD, 7-Aminoactinomycin D, AERD, Aspirin-exacerbated respiratory disease, CDR, Complementarity-determining region, CRSwNP, Chronic rhinosinusitis with nasal polyps, Fab, Antigen-binding fragment, FACS, Fluorescence-activated cell sorting, SAE, *Staphylococcus aureus* enterotoxin, SE, Staphylococcal enterotoxin, SpA, Staphylococcal protein A, SPR, Surface plasmon resonance, TCR, T-cell receptor, TSST-1, Toxic shock syndrome toxin 1, VH, Heavy-chain variable region, VL, Light-chain variable region

## Abstract

**Background:**

Chronic rhinosinusitis with nasal polyps is associated with local immunoglobulin hyperproduction and the presence of IgE antibodies against *Staphylococcus aureus* enterotoxins (SAEs). Aspirin-exacerbated respiratory disease is a severe form of chronic rhinosinusitis with nasal polyps in which nearly all patients express anti-SAEs.

**Objectives:**

We aimed to understand antibodies reactive to SAEs and determine whether they recognize SAEs through their complementarity-determining regions (CDRs) or framework regions.

**Methods:**

Labeled staphylococcal enterotoxin (SE) A, SED, and SEE were used to isolate single SAE-specific B cells from the nasal polyps of 3 patients with aspirin-exacerbated respiratory disease by using fluorescence-activated cell sorting. Recombinant antibodies with “matched” heavy and light chains were cloned as IgG_1_, and those of high affinity for specific SAEs, assayed by means of ELISA and surface plasmon resonance, were recloned as IgE and antigen-binding fragments. IgE activities were tested in basophil degranulation assays.

**Results:**

Thirty-seven SAE-specific, IgG- or IgA-expressing B cells were isolated and yielded 6 anti-SAE clones, 2 each for SEA, SED, and SEE. Competition binding assays revealed that the anti-SEE antibodies recognize nonoverlapping epitopes in SEE. Unexpectedly, each anti-SEE mediated SEE-induced basophil degranulation, and IgG_1_ or antigen-binding fragments of each anti-SEE enhanced degranulation by the other anti-SEE.

**Conclusions:**

SEEs can activate basophils by simultaneously binding as antigens in the conventional manner to CDRs and as superantigens to framework regions of anti-SEE IgE in anti-SEE IgE-FcεRI complexes. Anti-SEE IgG_1_s can enhance the activity of anti-SEE IgEs as conventional antibodies through CDRs or simultaneously as conventional antibodies and as “superantibodies” through CDRs and framework regions to SEEs in SEE–anti-SEE IgE-FcεRI complexes.

*Staphylococcus aureus* and its superantigens are implicated in the intense inflammatory processes of the upper and lower airways in patients with allergic diseases.^1^ These superantigens, in particular *Staphylococcus aureus* enterotoxins (SAEs), are strongly associated with chronic rhinosinusitis with nasal polyps (CRSwNP), particularly in the subpopulation of patients with aspirin-exacerbated respiratory disease (AERD), as well as those with allergic rhinitis, asthma, and atopic dermatitis.^2^, ^3^, ^4^, ^5^ SAEs are a family of structurally related proteins comprising different serological types, such as staphylococcal enterotoxin (SE) A, SEB, SEC, SED, and SEE (up to SEU) and toxic shock syndrome toxin 1 (TSST-1).^6^ SAEs are potent T-cell superantigens, causing polyclonal activation of up to 25% of certain T-cell populations by interacting with a common β-chain structural framework region in the T-cell receptor (TCR),^7^, ^8^ rather than the complementarity-determining region (CDR), which recognizes specific antigenic peptides bound to MHC. The activity of SEA and SED on B cells *in vitro* suggests that they can also act as B-cell superantigens by binding to the common structural framework regions in the immunoglobulin heavy-chain variable region (VH) domains shared by immunoglobulins with different CDRs,^9^, ^10^, ^11^ as previously shown for staphylococcal protein A (SpA).^12^, ^13^, ^14^ This might increase the polyclonality of the B-cell repertoire in patients with CRSwNP and allow *S aureus* to escape immune surveillance.^15^, ^16^

Nasal polyps in patients with CRSwNP are inflammatory outgrowths of the paranasal sinus mucosa, which are generally characterized by T_H_2 inflammation, local immunoglobulin production, and eosinophil infiltration driven by IL-5 and eotaxin.^17^, ^18^, ^19^, ^20^ Up to 100% of patients with AERD express anti-SAE IgEs in their nasal polyp homogenates and often have a higher prevalence of comorbid asthma and eosinophilic inflammation,^17^, ^21^, ^22^, ^23^ and IgEs from nasal polyps activate basophils in response to allergens and SEB *in vitro*.^15^ SEB acts as a human T-cell superantigen *in vivo* to cause the symptoms of atopic dermatitis.^24^ Basophils isolated from patients with atopic dermatitis with anti-SAE IgE in their sera, but not those from healthy control subjects, were responsive to SAEs.^25^ Although anti-SAE IgE can be detected in the circulation of patients with CRSwNP, B cells expressing this IgE are confined to the nasal mucosa, suggesting a role in sinonasal inflammation.^17^ Whether the SAEs bind to CDRs, framework regions, or both is addressed in the present work.

Antibody production in nasal polyps of patients with CRSwNP is driven by local activation and differentiation into plasma cells of B cells on exposure to various aeroallergens and microbial antigens.^26^, ^27^ Thus nasal polyps removed from patients with AERD provide a unique source of tissue to study how SAEs can shape the local antibody repertoire. In the first study of this kind, we used an efficient single-cell RT-PCR method to clone and express antibodies from single SAE-specific B cells isolated from the nasal polyps of 3 patients with AERD and tested their primary function in effector cell activation.

## Methods

### Patients' samples

Nasal polyps and sera were collected from 3 patients with AERD (HPK-014, HPK-016, and HPK-018) at the Royal National Throat, Nose and Ear Hospital, London, United Kingdom. The patients were male, with a mean age of 56 years. Only patient HPK-014 had positive skin prick test responses to aeroallergens. The ethical committee representing the Royal National Throat, Nose and Ear Hospital approved the study, and all patients provided written informed consent before the study commenced.

### Measurement of IgE to SAEs in sera and nasal polyp homogenates

Detailed methods for processing sera and nasal polyp homogenates are available in the Methods section in this article's Online Repository at www.jacionline.org. All sera from blood samples and homogenates from nasal polyps were assayed for total IgE and specific IgE to SEA, SEB, SEC1, SED, SEE, and TSST-1 by using the UniCAP system (Phadia, Uppsala, Sweden; see Table E1 in this article's Online Repository at www.jacionline.org).

### Single B-cell sorting by using fluorescence-activated cell sorting and single-cell RT-PCR of immunoglobulin cDNA

**Fig E1:**
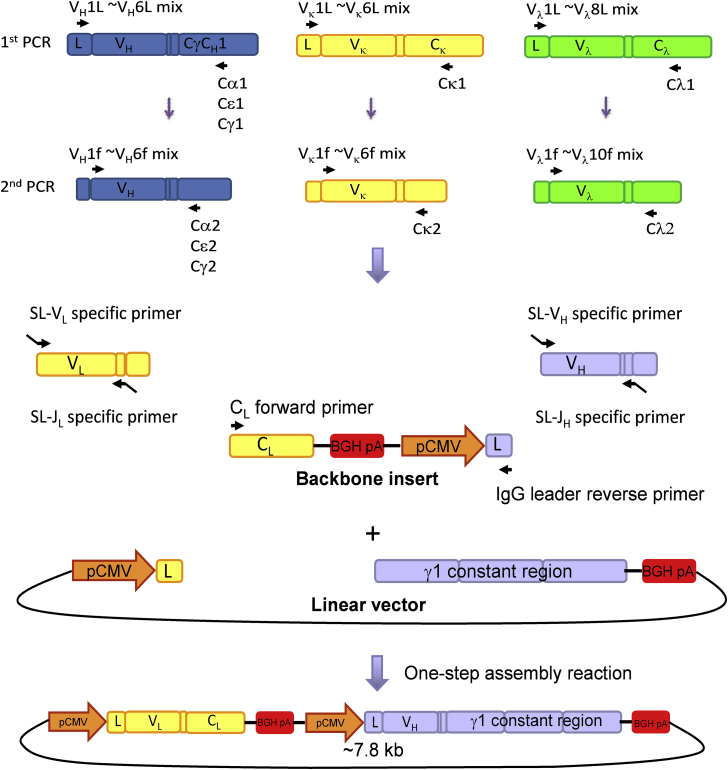
Cloning strategy for constructing recombinant IgG_1_ expression vector. Paired VH and VL regions from sorted single SAE-specific B cells were amplified by means of RT-PCR and sequenced as described in the Methods section in this article's Online Repository. The 15-bp homologous arm was introduced by means of PCR to permit cloning into pIgG_1_(κ) or pIgG_1_(λ) vectors by means of homologous recombination with the Seamless method, as described in the Methods section in this article's Online Repository. *C*_*L*_, Constant region of the light chain; *J*_*H*_, J region of the heavy chain; *J*_*L*_, J region of the light chain; *L*, leader; *SL*, seamless.

**Fig 1:**
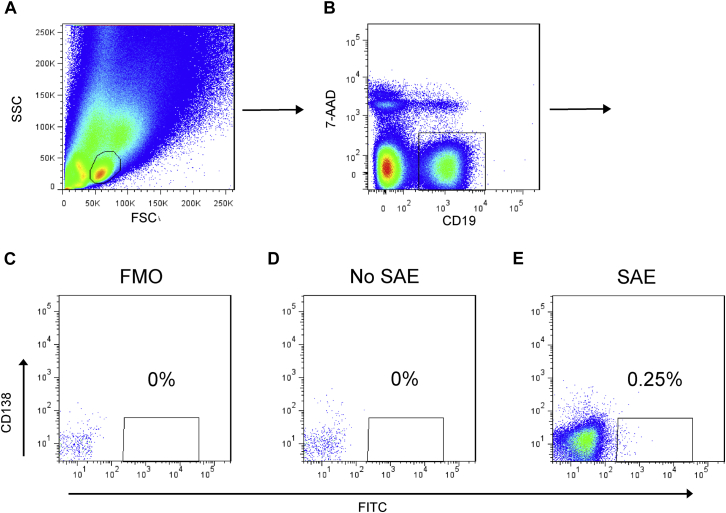
Isolation of SAE^+^ B cells from nasal polyps by means of FACS. Cells were labeled with a mixture of biotinylated SEA, SED, and SEE and phycoerythrin-coupled anti-CD138, allophycocyanin-coupled anti-CD19, and 7-AAD. **A,** The lymphocyte population was selected for size and granularity. *FSC*, Forward scatter; *SSC*, side scatter. **B,** Live B cells were identified as CD19^+^7-AAD^−^ cells. **C-E,** Fluorescence minus one *(FMO)* control (Fig 1, *C*) and no SAE control (Fig 1, *D*) were used to select SAE^+^CD19^+^CD138^−^7-AAD^−^ cells, which constituted 0.25% of the total B-cell population (Fig 1, *E*). The *No SAE* control indicates that the cells were stained with all fluorescent antibodies and streptavidin–fluorescein isothiocyanate *(FITC)* but without biotinylated SAEs, whereas FMO control indicates cells stained with all antibodies but without streptavidin-FITC.

Single-cell suspensions from frozen nasal polyp samples were prepared by 1 hour of digestion with 1 mg/mL hyaluronidase (Sigma-Aldrich, St Louis, Mo) and 1 U/mL Liberase TL (Roche, Mannheim, Germany) at 37°C. Samples were stained for CD19, CD138, SEA, SED, and SEE. SAE-specific single B cells were sorted by means of fluorescence-activated cell sorting (FACS) into 96-well PCR plates for cDNA synthesis and immunoglobulin expression cloning as IgG_1_ (Fig 1). Full details of the cloning and expression strategy are available in Fig E1 and Table E2 in this article's Online Repository at www.jacionline.org.

### Antibody cloning, expression, and purification

**Fig E2:**
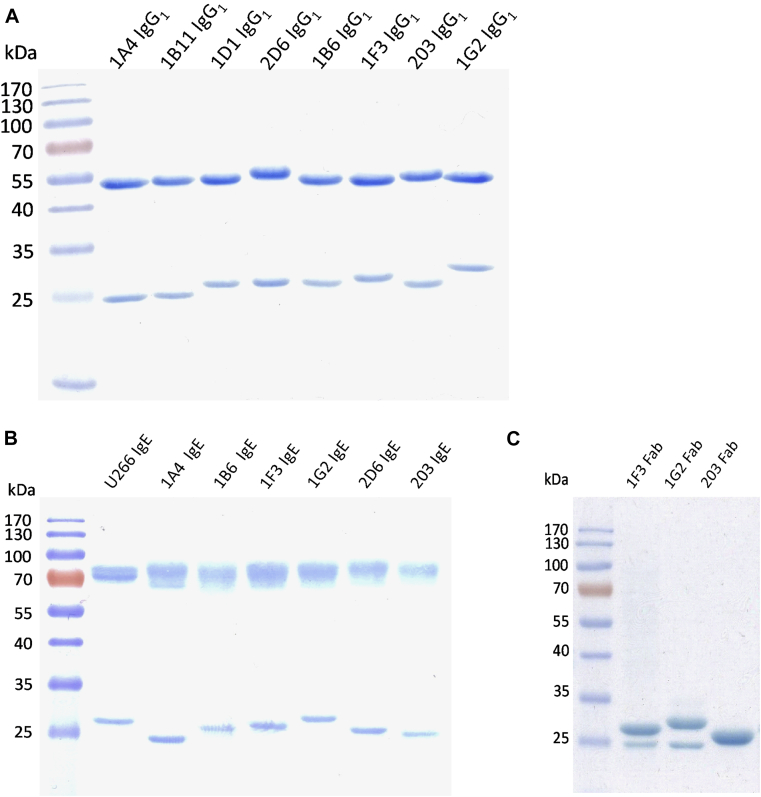
Purity of recombinant SAE-specific IgG_1_, IgE, and Fab antibodies. All recombinant SAE-specific IgG_1_**(A)**, IgE **(B)**, and Fab **(C)** antibodies were transiently expressed in Expi293F cells and purified by means of protein A, anti-IgE, or LambdaFabSelect affinity chromatography, respectively. Five micrograms of each antibody was fractionated by using 12% SDS-PAGE under reducing conditions before Coomassie Blue staining.

**Fig 2:**
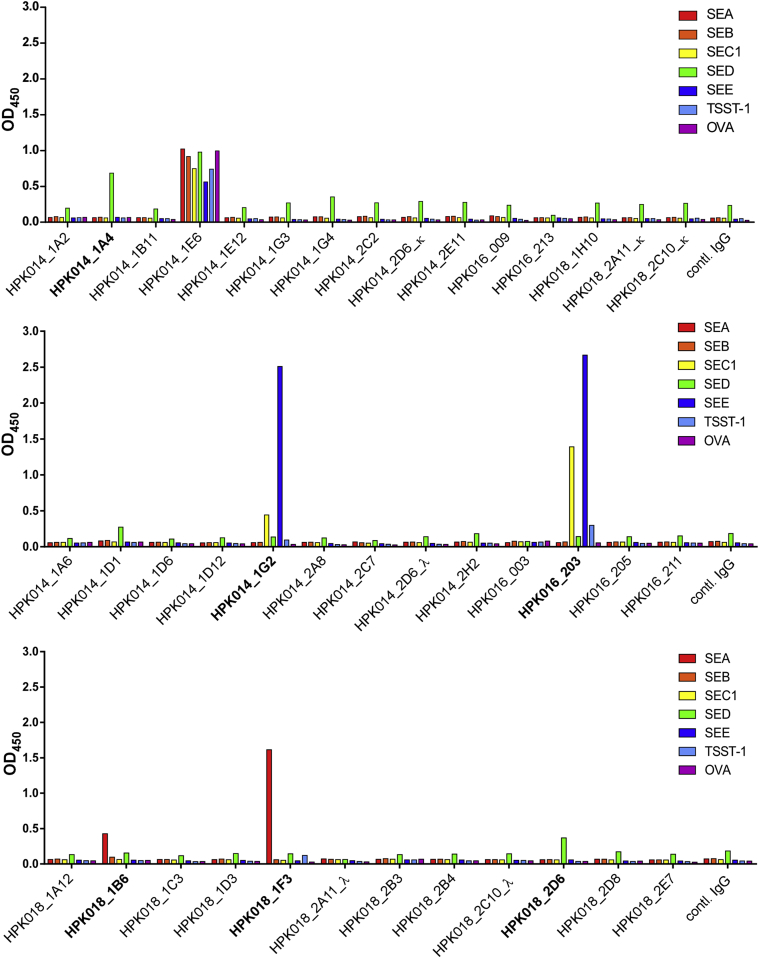
Six of the expressed antibodies exhibited SAE binding. The cleared supernatants collected from 293T cells, separately transfected with 40 IgG_1_ expression plasmids, were tested for reactivity with SEA, SEB, SEC1, SED, SEE, TSST-1, and ovalbumin *(OVA)* by means of ELISA. An antibody with specificity to a particular SAE was identified with an absolute OD value greater than that of the than control IgG. The antibodies selected for further studies are shown in boldface. Control IgG is a human IgG_1_ with VH4 and Vκ. We expected to select only high-affinity binders by using ELISA because of the low sensitivity of this assay.

SEA-, SED-, and SEE-specific antibodies were expressed in human embryonic kidney 293T cells (ATCC, Manassas, Va) as IgG_1_. Large-scale production of IgE, IgG_1_, and antigen-binding fragments (Fabs) was carried out with Expi293 cells (Invitrogen, Carlsbad, Calif). The IgG_1_ antibodies were purified by means of protein A–Sepharose (GE Healthcare, Pittsburgh, Pa), the IgE antibodies by omalizumab-coupled NHS-activated Sepharose (GE Healthcare), and the Fabs by LambdaFabSelect agarose (GE Healthcare). Anti-SAE IgG_1_ antibodies were identified in culture supernatants by means of routine ELISA (Fig 2), and their purity was estimated by means of SDS-PAGE under reducing conditions (see Fig E2 in this article's Online Repository at www.jacionline.org). Further details are available in the Methods section in this article's Online Repository.

### Sequence analysis of the monoclonal immunoglobulin genes

Immunoglobulin gene sequences were analyzed by using the IMGT/V-Quest tool,^28^ allowing identification of their clonal signatures (unique CDR3 sequences); germline V, D, and J genes; and somatic mutations. Sequences are available in GenBank (http://www.ncbi.nlm.nih.gov/genbank), and their accession numbers are listed in Table E3 in this article's Online Repository at www.jacionline.org.

### Surface plasmon resonance

Surface plasmon resonance (SPR) with a Biacore T200 instrument (GE Healthcare) was performed to determine binding specificity, kinetics, and affinity of the antigen-antibody interactions. Further details are available in the Methods section in this article's Online Repository.

### Basophil degranulation assay

Degranulation of cells of the rat basophilic cell line RBL SX-38, which stably expresses the α, β, and γ chains of human FcεRI,^29^ was used as a routine method to measure the functionality of the SAE-specific antibodies. Further details are available in the Methods section in this article's Online Repository.

## Results

### Cloning of anti-SAE antibodies

Three patients with AERD, HPK-014, HPK-016, and HPK-018, with titers of specific anti-SAEs in their serum or nasal polyp homogenate were studied (see Table E1). Specific IgEs to 6 common SAEs (SEA, SEB, SED, SEE, SEC, and TSST-1) were detected in all 3 homogenate samples and in the serum of patient HPK-018 and to 3 SAEs (SEA, SEE, and TSST-1) in the serum of patient HPK-14, whereas none were detected in the serum of patient HPK-016. Percentages of the anti-SAE IgEs were approximately 10-fold higher in the homogenates compared with the sera, ranging from 0.38% to 2.35% and 0.03% to 0.31%, respectively (see Table E1).

Viable SAE-positive B cells (SAE^+^CD19^+^CD138^−^ 7-aminoactinomycin D [7-AAD]^−^) identified by means of FACS (Fig 1) were sorted into individual wells of 96-well plates. SAE-bound B cells accounted for 0.18%, 0.08%, and 0.25% of the nasal polyp B-cell population of patients HPK-014, HPK-016, and HPK-018, respectively (see Table E4 in this article's Online Repository at www.jacionline.org). Matched immunoglobulin VH and light-chain variable region (VL; κ or λ) DNA pairs were amplified from the cDNAs of 37 SAE^+^CD19^+^CD138^−^ single B cells (18 from patient HPK-014, 6 from patient HPK-016, and 13 from patient HPK-018; see Fig E1). Of these 37 VH sequences obtained, 23 were from γ chains, and 14 were from α chains. No VH sequence was obtained from ε chains. The VH genes used in these 37 VH sequences consisted of 12 VH1 (32.43%), 13 VH3 (35.14%), 6 VH4 (16.22%), 5 VH5 (13.51%), and 1 VH6 (2.7%) sequences. Three (HPK-014_2D6, HPK-018_2A11, and HPK-018_2C10) of the 37 B cells expressed both a κ and a λ chain. Of 40 VL sequences obtained, 25 were from the λ chain, and 15 were from the κ chain. All 37 B cells represented different clones ascertained by their unique CDR-H3 sequences (BioProject ID: PRJNA347912; https://www.ncbi.nlm.nih.gov/bioproject). We expressed all 40 of the putative SAE-specific matched variable regions as human IgG_1_ antibodies.

### Identification and sequence analyses of anti-SAE antibodies

All 40 antibodies, expressed as IgG_1_, were assayed for the 6 SAEs detected in nasal polyp homogenates by means of ELISA. Only SEA-, SED-, and SEE-specific antibodies could be identified, which is consistent with screening the B cells for only these specificities. Two anti-SEA antibodies, HPK-018_1B6 (IgA_1_, *IGHV1-46*) and HPK-016_1F3 (IgG_1_, *IGHV1-69*); 2 anti-SED antibodies, HPK-014_1A4 (IgA_2_, *IGHV3-34*) and HPK-018_2D6 (IgA_1_, *IGHV3-49*); and 2 anti-SEE antibodies, HPK-014_1G2 (IgA_1_, *IGHV3-30*) and HPK-016_203 (IgG_4_, *IGHV5-51*), were identified (Fig 2). The germline immunoglobulin variable genes and isotypes used by those 6 antibodies are shown in Table E3. We refer to these antibodies below as the anti-SEA antibodies 1B6 and 1F3, the anti-SED antibodies 1A4 and 2D6, and the anti-SEE antibodies 1G2 and 203.

**Fig E3:**
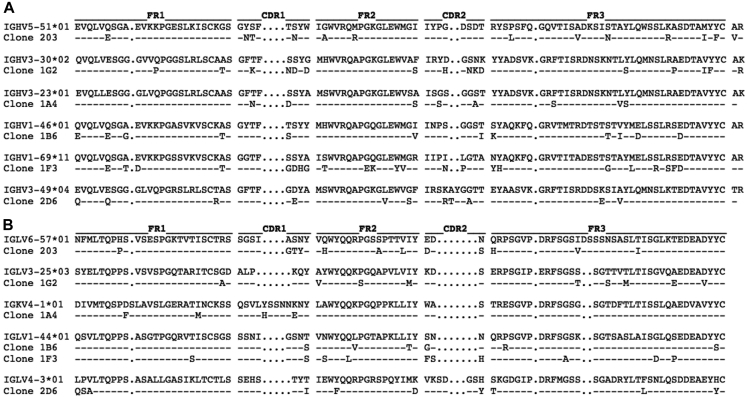
Amino acid sequence alignment for 6 cloned SAE-specific antibodies against germline sequences. The V genes encoding the VH **(A)** and VL **(B)** of SAE-specific antibodies were identified by using IMGT/V-Quest. Somatic hypermutations that resulted in amino acid changes are indicated.

The V(D)J rearrangement and CDR signatures of the 6 anti-SAE antibodies are shown in Table E3. A higher frequency of λ light chains (5/6) was found when compared with that in human peripheral blood B cells (1/5).^30^ Sequence alignments of 6 antibodies against their most closely related germline VH and VL gene sequences revealed a relatively high frequency of amino acid replacements in framework regions of VH and VL chains (see Fig E3 in this article's Online Repository at www.jacionline.org).

### Specificity and affinity of purified anti-SAE antibodies

**Fig 3:**
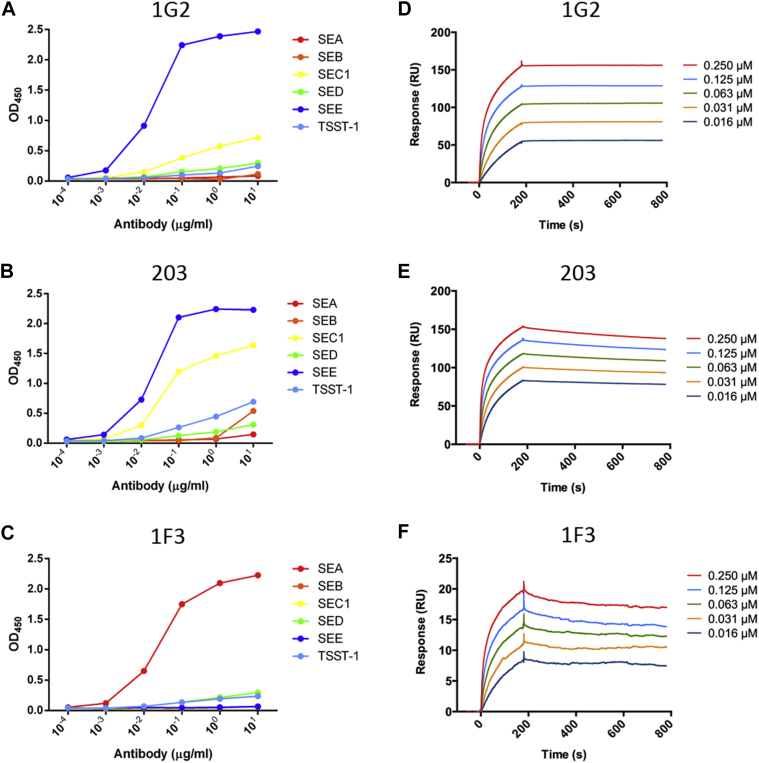
Specificity and high affinity of anti-SEE and anti-SEA antibodies. **A-C,** Recombinant IgG_1_ antibodies 1G2, 203, and 1F3 at indicated concentrations were tested for binding to SEA, SEB, SEC1, SED, SEE, and TSST-1 by using ELISA. **D-F,** Binding kinetics were characterized by means of SPR with biotin-labeled SEE or SEA immobilized on a streptavidin-coated sensor chip. Purified antibodies were injected at the indicated concentrations, followed by dissociation.

ELISA was used to determine the specificity of the purified recombinant IgG_1_ antibodies with the SAEs (Fig 3, *A-C*, and results not shown). The 1G2 and 203 antibodies were specific for SEE, although both displayed cross-reactivity with SEC1 (27% amino acid sequence homology with SEE) to a different extent rather than the more homologous SEA and SED (81% and 54% sequence homology with SEE, respectively).^31^ 1F3 was specific for SEA but was not cross-reactive with SEE. All 3 antibodies exhibited high affinity for their cognate SAEs, demonstrating low nanomolar binding affinities, as measured from the kinetics of interaction with specific SAEs by using SPR (Fig 3, *D-F*).

### Anti-SEE IgE mediates SEE-induced basophil degranulation

**Fig 4:**
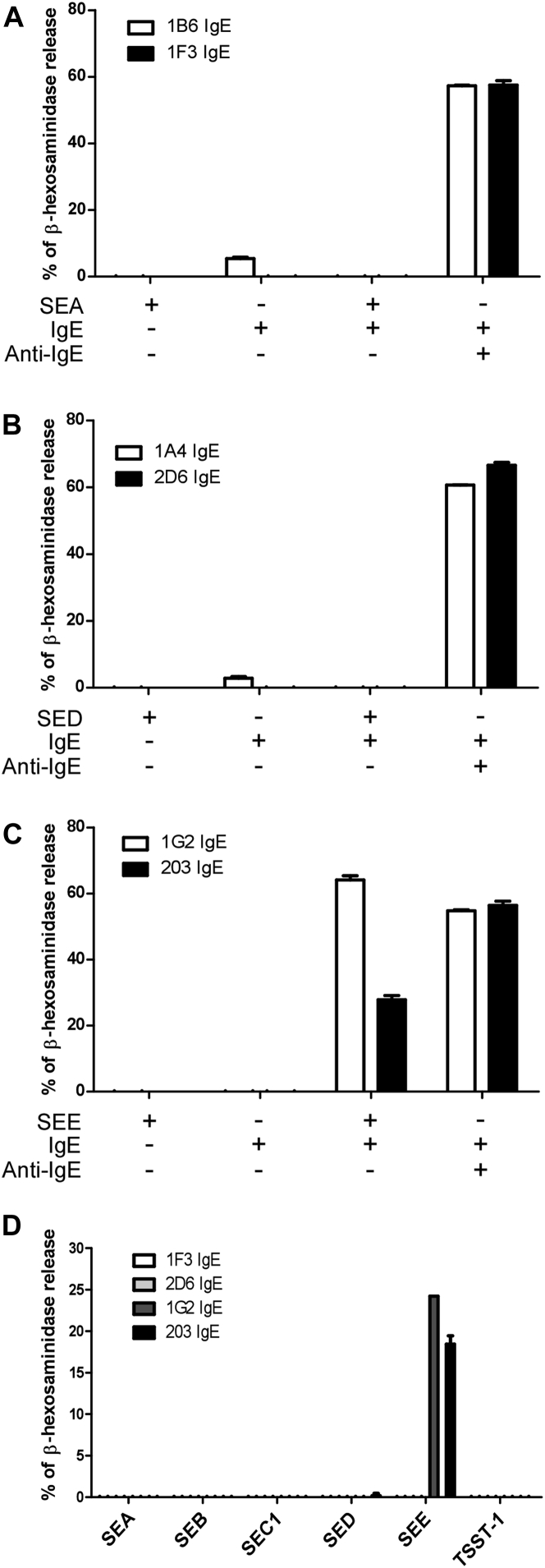
Anti-SAE IgEs mediate SAE-induced basophil degranulation. **A-C,** RBL SX-38 cells were sensitized with anti-SEA IgE 1B6 or 1F3 (Fig 4, *A*), anti-SED IgE 1A4 or 2D6 (Fig 4, *B*), or anti-SEE IgE 1G2 or 203 (Fig 4, *C*) and stimulated by the cognate SAE or anti-IgE as a positive control. **D,** RBL SX-38 cells were sensitized with the indicated anti-SAE IgEs and stimulated with the indicated SAEs. Degranulation was assayed based on β-hexosaminidase release.

The γ1 chain constant region of the IgG_1_ antibodies was substituted with the human ε constant region and the resulting IgEs were expressed and purified to examine the activities of the corresponding anti-SAE IgEs (see Fig E2, *B*). Basophil degranulation activities of the 6 anti-SAE IgEs in the presence of the cognate SAE are shown in Fig 4.

Conventional antigen binding through CDRs to 2 identical receptor-bound monoclonal IgE molecules, each of which recognizes a single epitope in a monomeric antigen, should not induce cross-linking of FcεRI on the surfaces of basophils to cause basophil degranulation. The anti-SEAs (Fig 4, *A*) and the anti-SEDs (Fig 4, *B*) behaved as expected, whereas both anti-SEE IgEs, 1G2 and 203, were active in this assay (Fig 4, *C*). Basophil activation would require either 2 identical epitopes in the SEE because it formed a dimer like SED^32^ or 2 independent epitopes in an SEE molecule if it were a monomer. A recent crystal structure of SEE in a complex with TCR revealed a monomeric structure.^33^ Thus we conclude that SEE has 2 distinct and nonoverlapping epitopes that explains this activity; this is illustrated and discussed below. SEC1 did not trigger 1G2 or 203 IgE-mediated basophil degranulation, although both show cross-reactivity to SEC1 (Fig 4, *D*).

### Anti-SEE antibodies bind to nonoverlapping epitopes on SEE

**Fig E4:**
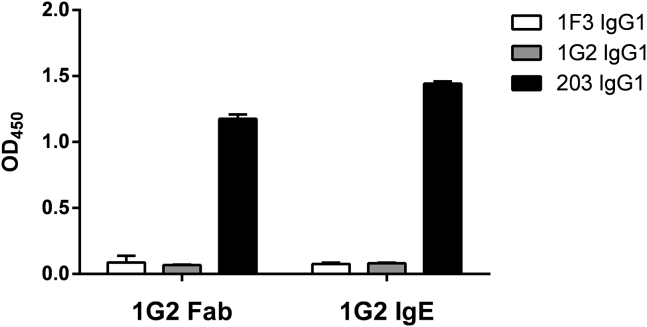
1G2 and 203 bind to distinct epitopes on SEE. Each well of a 96-well ELISA plate was coated with 1G2 Fab or IgE antibodies at 1 μg/mL (50 μL/well), followed by saturating 1G2 antibody binding sites with SEE at 1 μg/mL (100 μL/well). The 1F3, 1G2, and 203 IgG_1_ antibodies were added to each well, followed by incubation with peroxidase-conjugated goat anti-human IgG/Fc.

**Fig 5:**
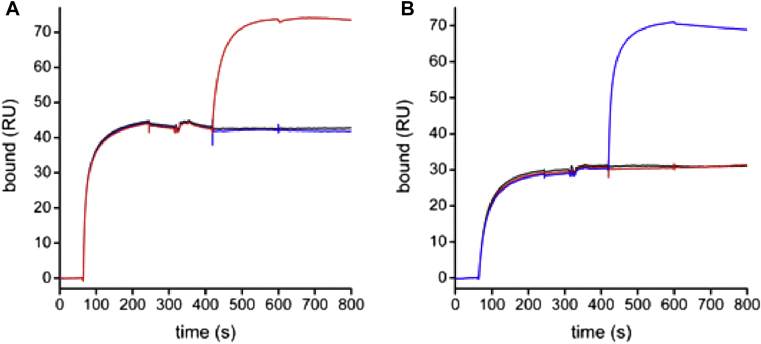
Anti-SEE antibodies bind to distinct nonoverlapping epitopes. **A,** 1G2 IgG_1_ (250 nmol/L) was injected onto a sensor chip coated with SEE for 3 minutes, immediately followed by a 3-minute injection of either running buffer *(black)*, 500 nmol/L 1G2 IgG_1_*(blue)*, or 100 nmol/L 203 IgG_1_*(red)*. **B,** After regeneration of the chip by using glycine-HCl (pH 2.5), the experiment was performed in reverse, with a 3-minute binding of 100 nmol/L 203 IgG_1_, followed by a 3-minute injection of either buffer *(black)*, 500 nmol/L 203 IgG_1_*(red)*, or 100 nmol/L 1G2 *(blue)*.

To map the epitopes of anti-SEE 1G2 and 203 antibodies on SEE, we carried out sandwich binding assays using ELISA and SPR. ELISA revealed that saturation of the 1G2 binding site, which was achieved through addition of SEE to immobilized 1G2 Fab and IgE, did not prevent binding of 203 IgG_1_ (see Fig E4 in this article's Online Repository at www.jacionline.org). Also, SPR revealed that binding of saturating concentrations of 1G2 IgG_1_ to immobilized SEE did not prevent the simultaneous binding of soluble 203 IgG_1_ (Fig 5, *A*). The reverse was also observed: after saturating all of the 203 binding sites on immobilized SEE, 1G2 IgG_1_ could still bind with high affinity (Fig 5, *B*). Both ELISA and SPR assays confirmed that the 2 anti-SEE antibodies bind to nonoverlapping epitopes on SEE.

### Anti-SEE IgG_1_ modulates IgE-mediated degranulation

**Fig 6:**
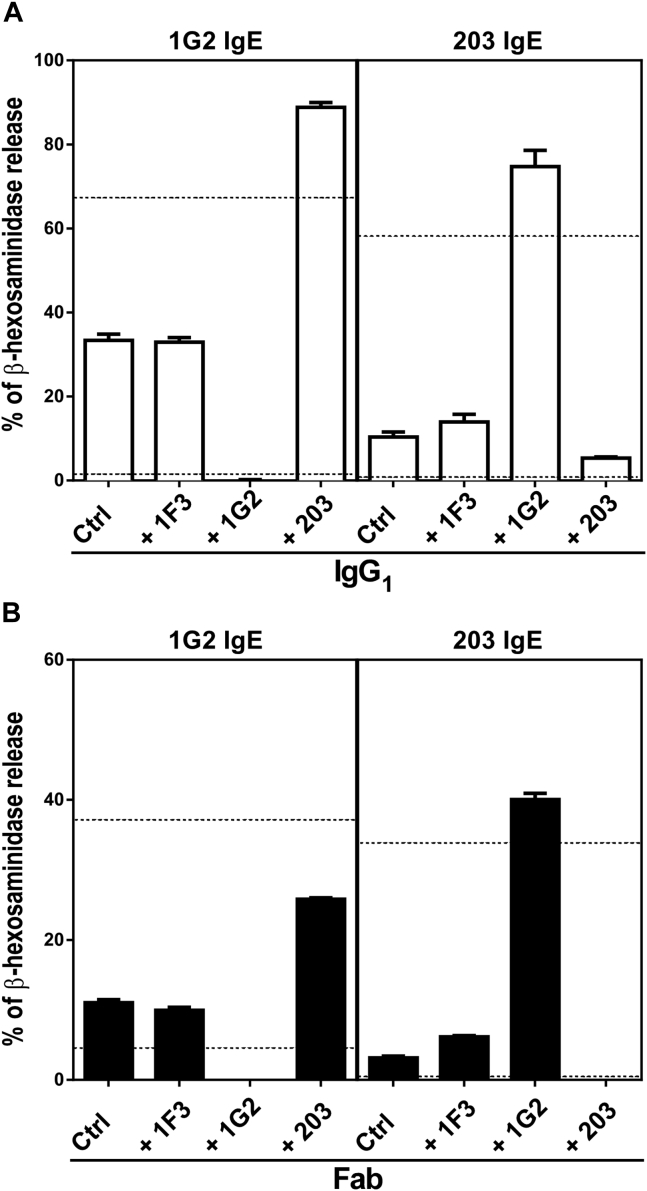
The unrelated anti-SEE IgG_1_ or Fab enhances anti-SEE IgE–mediated basophil degranulation. RBL SX-38 cells were sensitized with anti-SEE or anti-SEA IgE antibodies and stimulated with SEE in the presence of anti-SEE (+1G2/+203) or anti-SEA (+1F3) IgG_1_**(A)** or Fab **(B)**. *Dotted lines* represent positive (*upper*; anti-IgE) and negative (media only) controls. Degranulation was assayed based on β-hexosaminidase release.

**Fig 7:**
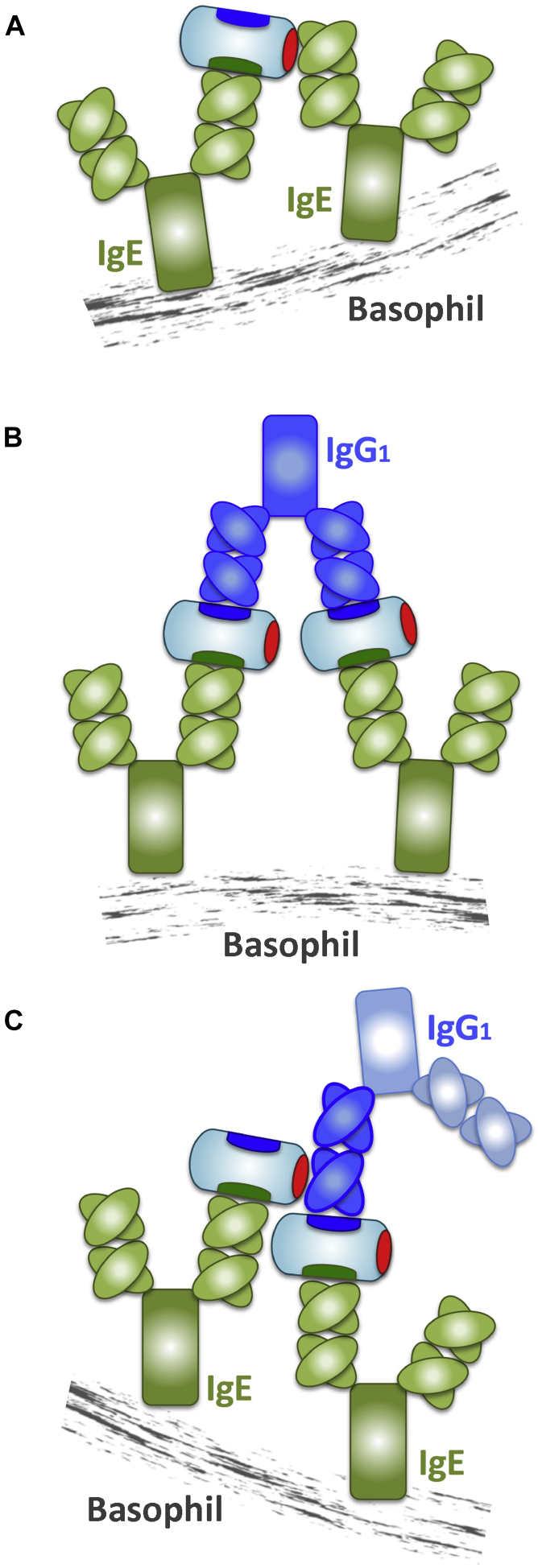
Schematic representation of proposed antibody and “superantibody” activities. **A,** A monomeric SEE molecule cross-links 2 FcεRI-bound IgE molecules on the surface of a basophil (receptor not shown) through 2 epitopes: the *green* site is recognized conventionally by the CDRs of the IgE antibody, and the *red* site interacts with the framework regions of the IgE V region in a superantigen manner. **B,** An IgG_1_ antibody that recognizes a nonoverlapping *blue* epitope cross-links 2 FcεRI-bound IgE molecules that recognize the *green* epitope; both *blue* and *green* epitopes are recognized conventionally by the CDRs. **C,** Because the IgG_1_ Fab (highlighted in *darker blue*) can also cross-link FcεRI-bound IgE, we propose that 2 monomeric SEE molecules can affect this: one is recognized conventionally through the 2 nonoverlapping (*green* and *blue*) epitopes by 2 unrelated anti-SEEs, and the other involves the superantigen site *(red)*. Although the cross-linking ability of the Fab reveals the interactions that underpin this proposed mechanism, the whole antibody can act not only in the manner depicted in Fig 7, *B*, but also that shown in Fig 7, *C*. This antibody, exhibiting the ability to recognize 2 SEE molecules simultaneously through both CDRs and framework regions, we call a “superantibody.”

The different anti-SEE isotypes, IgE and IgG_1_, were tested in pairwise combinations in basophil degranulation assays to characterize their interplay. As expected, because of competition for epitope binding to SEE, the 1G2 IgG_1_ inhibited SEE-induced degranulation of basophils sensitized with the 1G2 IgE, and the 203 IgG_1_ inhibited degranulation of basophils sensitized with the 203 IgE (Fig 6, *A*).^34^, ^35^ As also expected, the IgG_1_ that recognized the nonoverlapping epitope on IgE enhanced degranulation (Fig 7, *A*) by cross-linking 2 SEE molecules bound to adjacent receptor-bound IgE molecules; this is illustrated below in Fig 5, *B*. The 1F3 antibody, which is specific for SEA, had no such effects, demonstrating that antigen specificity is crucial for the modulation of IgE activity by IgG_1_.

### Paradoxical enhancing activity of anti-SEE Fabs

We repeated the pairwise assays by using the 2 anti-SEE IgG_1_ Fabs (Fig 6, *B*). As expected, the Fabs exhibited the same inhibitory activity as the whole IgG_1_ antibodies (because of competitive inhibition) when Fab and IgE were of the same specificity. Cross-linking of adjacent IgE-SEE complexes on the basophil surface cannot occur with a monomeric Fab, even one that binds to the nonoverlapping epitope. Paradoxically, the Fab recognizing the nonoverlapping epitope retains the enhancing activity of the whole IgG_1_ (Fig 6, *B*).

Fig 7, *C*, illustrates the mechanism by which receptor cross-linking can occur. We suggest that high-affinity binding of receptor-bound anti-SEE IgE to SEE through its CDRs to the epitope represented in green (Fig 7, *C*) facilitates subsequent low-affinity binding through a site on SEE, which is represented in red, that binds to a framework region of an adjacent IgG (or Fab) that recognizes a nonoverlapping SEE epitope, which is represented in blue. We call such an antibody, in principle of any isotype that acts synergistically to enhance IgE effector functions, a “superantibody.”

## Discussion

Several human mAbs against specific allergens have been obtained from allergic patients by means of phage display,^36^, ^37^, ^38^, ^39^, ^40^ EBV transformation of PBMCs,^41^ or single-cell RT-PCR.^34^ In addition, many groups have generated anti-SEB mAbs by using either mouse hybridomas or phage display and pursued their use as therapeutic agents.^42^, ^43^ In earlier work we produced a recombinant allergen (Phl p 7)–specific antibody with the native (“matched”) heavy and light chain by using single-cell PCR.^34^ To the best of our knowledge, the present work is the first report of recombinant anti-SAE antibodies containing matched heavy and light chains cloned by using single-cell RT-PCR. We produced 6 anti-SAE antibodies that bind to SAEs with high affinity: 2B6 and 1F3 for SEA, 1A4 and 2D6 for SED, and 1G2 and 203 for SEE. The VH regions of 1F3 and 203 were derived from single IgG-expressing B cells and 1B6, 1A4, 1G2, and 2D6 from single IgA-expressing B cells, which were present in nasal polyps from 3 patients with CRSwNP (see Table E3). The antibodies were first expressed as IgG_1_ and then converted to IgE and Fab.

We began with the hypothesis that SAEs can act as B-cell superantigens, as well as T-cell superantigens. Indeed, this suggestion has often been made before. It is consistent with the evidence that SEA and SED bind weakly to immunoglobulins bearing VH3 and VH4, respectively.^10^, ^11^ Several authors have demonstrated the overabundance of VH5 in IgE, suggesting that such a B-cell superantigen can recognize members of this VH family.^44^, ^45^, ^46^, ^47^ Low-affinity and promiscuous binding to the TCR variable β (Vβ) chains are characteristics of T-cell superantigens, which include SEA, SED, and, notably, SEE.^7^, ^8^ SEE binds to 6 different human Vβ chains.^48^ We have shown that SEE can indeed behave as a B-cell superantigen by interacting with IgE molecules both conventionally through CDRs and unconventionally through framework regions (Fig 7, *A*).

ELISA assays used to select the 6 antibodies described in the present study (Fig 3) would not have detected weak binding of SAEs to the framework regions. However, strong binding of SAEs to the CDRs in the context of a cell (Fig 2) would facilitate weak binding to the framework regions because of an increase in avidity. Binding of SAEs to the framework regions as well as CDRs of BCRs can contribute to somatic hypermutation and affinity maturation of antibodies. Coker et al^46^ found 75% of mutational hot spots in the framework regions of IgE and IgA expressed by B cells in the nasal mucosa, which are often exposed to *S aureus* commensal infections.^46^ Superantigen-driven selection at the stage of local affinity maturation might explain the high frequency of mutations in the framework regions of our cloned anti-SAEs (see Fig E3).

**Fig E5:**
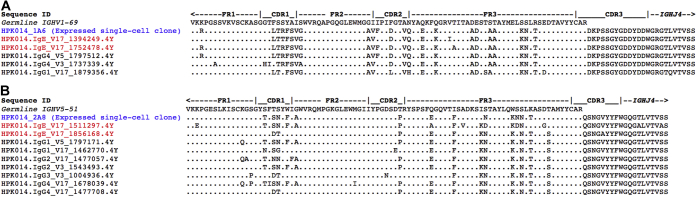
Expressed single-cell IgG_1_ clones with IgE relatives identified by using next-generation sequencing (NGS) of B-cell repertoire. The immunoglobulin VH gene repertoire in the nasal polyps from patient HPK-014 was captured on the Illumina 2x300 MiSeq System and bioinformatically analyzed (manuscript in preparation). The sequences in the HPK-014 NGS data set were aligned with the recombinant IgG_1_ antibodies expressed from 18 FACS-sorted SAE^+^ B cells (SAE^+^CD19^+^CD138^−^7-AAD^−^), and their clonal relatedness was determined by using clone-specific CDR3 DNA motifs. Two clonal families were identified, showing 2 expressed recombinant IgG_1_ antibodies (A: HPK-014_1A6 and B: HPK-014_2A8; Sequence ID in *blue*) related to IgE (sequence ID in *red*) and other IgG mutants in the HPK-014 NGS data set. For illustration purposes, not all members of the clones isolated by using NGS are shown.

Due to the real-world limitations of the experimental methods used in this study, we have not obtained definitive evidence for the proposed interactions (Fig 7). First, IgE-expressing antibodies were not among the 40 recombinant antibodies we expressed; this was not surprising because of the extremely low abundance of IgE- compared with IgG- and IgA-expressing B cells.^49^ Second, although we have shown that IgG-expressing B cells undergo local switching to IgE in nasal polyps from patients with CRSwNP and in the respiratory tract mucosa in patients with allergic diseases,^50^, ^51^, ^52^, ^53^ 1G2 and 203 antibodies came from different patients. We would have needed IgE related to at least 1 of them and both antibody isotypes from the same subject to prove the existence of superantibodies in this AERD cohort. Nevertheless, in unpublished work using next-generation sequencing of B-cell repertoires in nasal polyps, we have observed as many as 17/10,000 IgE sequences related to IgG or IgA. In the B-cell repertoire of patient HPK-014, we were able to identify IgE relatives of 2 recombinant IgG_1_ antibodies (A: HPK-014_1A6 and B: HPK_014 2A8; see Fig E5 in this article's Online Repository at www.jacionline.org), although these 2 IgG_1_ clones were not among the 6 clones that exhibited high affinity for the SAEs (Fig 3).

One of the antibodies, 203, was originally an IgG_4_ and, interestingly, expresses *IGHV5-51*, the only of 2 VH5 family members that is overexpressed in IgE (Wu et al, unpublished results) and a candidate for binding in a superantigen-like manner.^46^ The other antibody, 1G2, was originally an IgA_1_ and expresses a member of the VH3 family (*IGHV3-30*, see Table E3). The CDRs differ between 203 and 1G2, and therefore it might be that SEE binds promiscuously to both VH5 and VH3 framework regions. Structural studies are required to define the epitopes in SEE.

It was previously shown by using x-ray crystallography that SpA is recognized by a rheumatoid factor antibody in a superantigen-dependent manner, in which SpA interacts with the framework residues of the VH3 domain.^14^ We believe the anti-SEE superantibodies presented here might be the first antibodies shown to have both conventional antigen- and superantigen-binding sites and thus to have superantibody activity.

The ability of the anti-SEE IgG_1_ and its Fabs to inhibit the activity of basophils sensitized by the related IgE resembles the blocking activities of IgG_4_ or IgA in specific allergen immunotherapy.^34^, ^35^, ^54^ Blocking IgG antibodies have been developed by many groups for therapeutic use as antitoxins. Nearly all antitoxin antibodies and all isolated anti-SEB antibodies are blocking, as opposed to enhancing, antibodies.^42^, ^43^, ^55^ SAE vaccination has been investigated, with encouraging success for efficacy in treating infections with *S aureus*.^56^, ^57^ Whether anti-SEE IgGs from the present study might be similarly efficacious could be determined, but a combination of anti-SAEs might be even more efficacious than any single anti-SAE.

To the best of our knowledge, 1G2 and 203 are the first examples of enhancing anti-SAE antibodies. However, there is a precedent in the case of the antibody BAB2 against a dominant birch pollen allergen, Bet v 1.^58^ BAB2 and, notably, its Fab enhanced binding of Bet v 1 to IgE and caused immediate-type hypersensitivity reactions in human skin. The authors suggested that enhancing antibodies could account for the failure of specific allergen immunotherapy.^58^, ^59^, ^60^ Although enhancing antibodies are relatively rare, they might be important because their activity might “win” in competition with an excess of blocking antibodies.

In previous studies it has been demonstrated that certain antibodies against a specific allergen are more frequently observed than others in atopic patients; such allergens, that is, Der p 1 and Der p 2 in patients with house dust mite allergy, are said to be dominant allergens.^61^, ^62^ These allergens usually have multiple epitopes, allowing more extensive cross-linking of the IgE-FcεRI complexes on effector cells. In this case noncompetitive allergen-specific IgG or IgA, which are likely present in much higher concentrations than IgE, might be able to enhance cell activation if the configuration of the 2 sites and the resulting distance between cross-linked complexes is favorable (Fig 7, *B*).

SEE was first documented in a case of food poisoning.^63^ It was later identified as a 26.4-kDa single-chain protein belonging to the group III superantigens (the SEA superfamily), having strong homology to SEA and SED, as stated above. SEE has been shown to be a potent enterotoxin and a polyclonal activator of T cells^64^, ^65^ and binds promiscuously to multiple human TCR Vβ chains.^48^ Anti-SEE IgE was demonstrated in 16.7% of nasal polyps compared with 0% of control tissue, as well as in all nasal polyp samples from all 3 patients with CRSwNP in the present study.^17^ Sequence comparison of SEA, SEC1, SED, and SEE and inspection of the SEA and SEE crystal structures^32^, ^33^ suggest that certain surface-exposed residues could alter the electrostatic potential or shape of the enterotoxin surface, rendering SEE and SEC1 different from SEA and SED and thereby contributing to the cross-reactivity of the 203 antibody.

Basophils were activated to release granular mediators by allergens and SEB when sensitized by IgE in homogenates from the nasal polyps of patients with CRSwNP *in vitro*,^15^ indicating the functionality of IgE antibodies in nasal polyps. Thus the putative anti-SEE IgEs 1G2 and 203 in patients HPK_014 and HPK_016, together with the potential IgG_4_ or IgA_1_ precursors, could have activated a variety of effector cells and contribute to the proinflammatory environment in the patients' nasal polyps. This is compatible with evidence from the work of Gevaert et al^66^ showing that omalizumab is therapeutic in patients with CRSwNP, although an earlier study did not show efficacy.^67^ It should also be noted that IgG and IgA superantibody activities are independent of isotypes.

In summary, our results provide proof of concept that SAES can act as B-cell superantigens and that anti-SAE antibodies of any isotype can behave as superantibodies. We have isolated antibodies with potential superantibody activity from nasal polyps of patients with AERD, a highly inflammatory T_H_2 disease. However, similar antibodies can occur in patients with allergic diseases and asthma associated with commensal *S aureus* infections and dominant allergens.

**Clinical implications**

Anti-SAE IgE antibodies alone or together with IgG_1_ can contribute to the pathogenesis of CRSwNP and allergic disease.

## Figures and Tables

**Table E1:** SAE-specific IgEs in serum and nasal polyp homogenate

Patient ID	Total IgE	Serum						Total IgE	Nasal polyp homogenate					
		SEA	SEB	SED	SEE	SEC	TSST-1		SEA	SEB	SED	SEE	SEC	TSST-1
HPK-014	89.3	0.17∗ (0.19%)†	—	—	0.28 (0.31%)	—	0.16 (0.18%)	26.1	0.25 (0.96%)	0.12 (0.46%)	0.1 (0.38%)	0.13 (0.5%)	0.13 (0.5%)	0.21 (0.8%)
HPK-016	53.2	—	—	—	—	—	—	8.1	0.11 (1.36%)	0.11 (1.36%)	0.08 (0.99%)	0.09 (1.11%)	0.11 (1.36%)	0.19 (2.35%)
HPK-018	317	0.09 (0.03%)	0.81 (0.26%)	0.78 (0.25%)	0.13 (0.04%)	0.05 (0.02%)	0.07 (0.02%)	48	0.59 (1.23%)	0.24 (0.5%)	0.31 (0.65%)	0.27 (0.56%)	0.32 (0.67%)	0.28 (0.58%)

∗ Kilounits per liter was introduced to express the level of IgE.

† Percentage of total IgE was the percentage of each SAE-specific IgE in total IgE.

**Table E2:** Primers used for antibody cloning

Primer	Sequence
V_H_ cloning PCR	
SL-V_H_1/3/5f	gttgctacgcgtgtcCTGAGCSAGGTGCAGCTGGTGSAGTC
SL-V_H_1-3f	gttgctacgcgtgtcCTGAGCCAGGTCCAGCTTGTGCAGTC
SL-V_H_1-18f	gttgctacgcgtgtcCTGAGCCAGGTTCAGCTGGTGCAGTC
SL-V_H_1-24f	gttgctacgcgtgtcCTGAGCCAGGTCCAGCTGGTACAGTCTG
SL-V_H_2f	gttgctacgcgtgtcCTGAGCCAGGTCACCTTGARGGAGTCTG
SL-V_H_3-23f	gttgctacgcgtgtcCTGAGCGAGGTGCAGCTGTTGGAGTCT
SL-V_H_4f	gttgctacgcgtgtcCTGAGCCAGSTGCAGCTGCAGGAGT
SL-V_H_4-34f	gttgctacgcgtgtcCTGAGCCAGGTGCAGCTACARCAGTGG
SL-V_H_6f	gttgctacgcgtgtcCTGAGCCAGGTACAGCTGCAGCAGTCA
SL-V_H_7f	gttgctacgcgtgtcCTGAGCCAGGTGCAGCTGGTGCAAT
V_κ_ cloning PCR	
SL-V_κ_1f	ggctcccaggtgcacgatgtgACATCCAGWTGACCCAGTCTCC
SL-V_κ_2f	ggctcccaggtgcacgatgtgATATTGTGATGACCCAGACTCCACTCT
SL-V_κ_3f	ggctcccaggtgcacgatgtgAAATTGTGTTGACRCAGTCTCCAG
SL-V_κ_4f	ggctcccaggtgcacgatgtgACATCGTGATGACCCAGTCTC
SL-V_κ_5f	ggctcccaggtgcacgatgtgAAACGACACTCACGCAGTCTC
SL-V_κ_6f	ggctcccaggtgcacgatgtgAAATTGTGCTGACTCAGTCTCCA
V_λ_ cloning PCR	
SL-V_λ_1-40/50/51f	tccttgcttatgggtccggaGTGGATTCTCAGTCTGTGYTGACGCAGCC
SL-V_λ_1-36/44/47f	tccttgcttatgggtccggaGTGGATTCTCAGTCTGTGCTGACTCAGCCA
SL-V_λ_2f	tccttgcttatgggtccggaGTGGATTCTCAGTCTGCCCTGACTCAGCC
SL-V_λ_3f	tccttgcttatgggtccggaGTGGATTCTTCCTATGAGCTGACWCAGCCA
SL-V_λ_3-19f	tccttgcttatgggtccggaGTGGATTCTTCTTCTGAGCTGACTCAGGAC
SL-V_λ_4/5/9f	tccttgcttatgggtccggaGTGGATTCTCAGSCTGTGCTGACTCAGCC
SL-V_λ_4-60/69f	tccttgcttatgggtccggaGTGGATTCTCAGYCTGTGCTGACTCAATC
SL-V_λ_6f	tccttgcttatgggtccggaGTGGATTCTAATTTTATGCTGACTCAGCCC
SL-V_λ_7/8f	tccttgcttatgggtccggaGTGGATTCTCAGRCTGTGGTGACYCAGGAG
SL-V_λ_10f	tccttgcttatgggtccggaGTGGATTCTCAGGCAGGGCTGACTCAGCC
J_H_ cloning PCR	
SL-J_H_1/4/5r	gatgggcccttggtgctagcTGAGGAGACGGTGACCAGG
SL-J_H_2r	gatgggcccttggtgctagcTGAGGAGACAGTGACCAGGGT
SL-J_H_3r	gatgggcccttggtgctagcTGAAGAGACGGTGACCATTGTC
SL-J_H_6r	gatgggcccttggtgctagcTGAGGAGACGGTGACCGTG
J_κ_ cloning PCR	
SL-J_κ_1r	tgcagccaccgtacgTTTGATTTCCACCTTGGTCCCT
SL-J_κ_2r	tgcagccaccgtacgTTTGATCTCCAGCTTGGTCCCT
SL-J_κ_3r	tgcagccaccgtacgTTTGATATCCACTTTGGTCCCA
SL-J_κ_4r	tgcagccaccgtacgTTTGATCTCCACCTTGGTCCCT
SL-J_κ_5r	tgcagccaccgtacgTTTAATCTCCAGTCGTGTCCCTT
J_λ_ cloning PCR	
SL-J_λ_1r	ggccttgggctgacctaggACGGTGACCTTGGTCC
SL-J_λ_2/3r	ggccttgggctgacctaggACGGTCAGCTTGGTCC
SL-J_λ_6r	ggccttgggctgacctaggACGGTCACCTTGGTGC
SL-J_λ_7r	ggccttgggctgacctaggaCGGTCAGCTGGGTGC
Backbone insert cloning PCR	
SL-C_κ_+	CGTACGGTGGCTGCACCATC
SL-C_λ_+	GGTCAGCCCAAGGCCAACC
SL-IgG Leader-	GACACGCGTAGCAACAGCGA

Homologous sequences to the bases at one end of the adjacent DNA fragment are displayed in lower case.

**Table E3:** Germline immunoglobulin variable gene and CDR3 sequences of cloned VH and VL pairs in the SAE-specific antibodies

Clone name	Isotype	Accession (VH)	*IGHV*	*IGHD*	*IGHJ*	CDR-H3 amino acids	Accession (VL)	*IGK(L)V*	*IGK(L)J*	CDR-L3 amino acids
HPK-014_1A4	A (α2)	KT369789	3-23	3-9	3	CAKKRRYDAVTGYLFDMW	KT369790	4-1 (κ)	2	CQQFYSPAPYTF
HPK-014_1G2	A (α1)	KT369791	3-30	6-13	4	CARDRAQGIVAAAGTSFGYW	KT369792	3-25 (λ)	2	CQSGDTSGFDPDVAF
HPK-016_203	G (γ4)	KT369793	5-51	2-15	4	CVRHCSGGRCSSAPLDSW	KT369794	6-57 (λ)	3	CLSHDTTNFVF
HPK-018_1B6	A (α1)	KT369795	1-46	2-8	3	CAYWGADGFDIW	KT369796	1-44 (λ)	3	CAVWDDSLSAWVF
HPK-018_1F3	G (γ1)	KT369797	1-69	3-9	4	CARGMTGYVGAYDYW	KT369798	1-44 (λ)	3	CASWDDRLNGPVF
HPK-018_2D6	A (α1)	KT369799	3-49	2-2	4	CTTYCSSTSCQIDYW	KT369800	2-14 (λ)	1	CSSYTGSSGYVV

The VH and VL DNA sequences of each clone are available in GenBank (http://www.ncbi.nlm.nih.gov/genbank), and accession numbers are indicated in the table.

*IGHV*, Immunoglobulin heavy-chain variable region; *IGKV*, immunoglobulin kappa-chain variable region; *IGLV*, immunoglobulin lambda-chain variable region.

**Table E4:** Cell populations of nasal polyp tissues

	HPK-014	HPK-016	HPK-018
Lymphocytes∗	10.1% (255,337)	7.42% (157,133)	17.2% (313,277)
Live cells (7-AAD^−^)†	94.3% (239,878)	87.1% (136,067)	95.3% (297,159)
Live B cells (CD19^+^7-AAD^−^)†	20.7% (52,717)	21.1% (33,024)	29.4% (91,607)
SAE^+^ cells (SAE^+^CD138^−^)‡	0.18% (92)	0.08% (26)	0.25% (225)
Plasma cells (SAE^−^CD138^+^)‡	2.98% (1,573)	0.1% (34)	0.37% (339)

∗ 164% of total nasal polyp cells.

† 165% of lymphocytes.

‡ 166% of total live B cells.

## Methods

### Preparation of sera and nasal polyp homogenates

Blood samples were allowed to clot at room temperature for 1 hour and centrifuged at 2000*g* for 20 minutes, and serum was separated and stored at −70°C. PBS containing protease inhibitor cocktail (Roche) was added to snap-frozen nasal polyp specimens at 1 mL per 0.1 g of tissue. Tissues were homogenized with a TissueLyser (Qiagen, Hilden, Germany) for 2 minutes at 30 Hz until disrupted, homogenates were centrifuged at 15,000*g* for 20 minutes at 4°C, and supernatants were collected and stored at −70°C.

### Single cell sorting by using FACS

After dispersion by means of enzymatic digestion of nasal polyps, cells were washed with complete RPMI medium (10% FBS in RPMI medium) to remove the enzymes, filtered through a 40-mm cell strainer, and finally resuspended at a concentration of 1 × 10^6^ cells/mL in FACS buffer (5% normal goat serum [Invitrogen, Carlsbad, Calif] and 2 mmol/L EDTA in PBS). Then cells were stained with phycoerythrin-coupled anti-CD138 (BioLegend, San Diego, Calif), allophycocyanin-coupled anti-CD19 (BD Biosciences, San Jose, Calif), 7-AAD (eBioscience, San Diego, Calif), and biotinylated SAEs (Toxin Technology, Sarasota, Fla) in FACS buffer. Biotinylated SEA, SED, and SEE were simultaneously used for labeling cells each at a concentration of 10 μg/mL and detected by means of FACS with streptavidin–Alexa Fluor 488 (Invitrogen) at a dilution of 1:1000.

### Single-cell RT-PCR

Single SAE^+^CD19^+^CD138^−^7-AAD^−^ B cells were directly sorted into 96-well PCR plates (Fermentas, Vilnius, Lithuania) containing 18 μL/well of ice-cold 1× RT buffer (Invitrogen) containing 0.1% Triton-X 100, 10 mmol/L dithiothreitol, 300 ng of random hexamers (Invitrogen), 0.5 mmol/L dNTP mix (Fermentas), and 40 U of Ribolock RNase Inhibitor (Fermentas) on a FACSAria II cell sorter (BD Biosciences). Fifty units of Superscript III reverse transcriptase (Invitrogen) was added, and then reverse transcription was performed at 42°C for 10 minutes, 25°C for 10 minutes, 50°C for 60 minutes, and 85°C for 5 minutes.

Primer nucleotide sequences to cover all known V-region genes and reverse primers specific for κ and λ constant regions were as previously described.^E1^ Reverse primers for γ, α, and ε constant regions were Cγ1 (5′-GGAAGGTGTGCACGCCGCTGGTC-3′), Cε1 (5′-GGTTTTGTTGTCGACCCAGTCTG-3′), and Cα1 (5′-TGGGAAGTTTCTGGCGGTCACG-3′) as primary primers for the first-round PCR and Cγ2 (5′-GTTCGGGGAAGTAGTCCTTGAC-3′), Cα2 (5′-GTCCGCTTTCGCTCCAGGTCACACT-3′), and Cε2 (5′-TGGCATAGTGACCAGAGAGCGTG-3′) as nested primers for the second-round PCR, respectively. All PCRs were carried out by proofreading Phusion DNA polymerase (Fermentas). Second-round PCR products were sequenced after purification by using the QIAquick PCR Purification Kit (Qiagen).

### Recombinant antibody cloning and expression

Purified second-round PCR products were used as a PCR template for amplifying DNA inserts by V-region– and J-region–specific primers containing an overhang sequence (Table E2), which are homologous to the bases at one end of adjacent DNA fragments of pIgG_1_(κ) or pIgG_1_(λ) vectors.^E2^ Purified *Dra*III/*Nhe*I- and *Bsp*EI/*Nhe*I-digested DNA fragments from pIgG_1_(κ) and pIgG_1_(λ) vectors were used as a template for amplifying “backbone insert” by CL forward primers, SL-Cκ+ or SL-Cλ+, and the IgG leader reverse primer SL-IgG Leader- (Fig E1 and Table E2), respectively. All PCR products above were synthesized by using Phusion DNA polymerase and purified with the QIAquick PCR Purification Kit. The linear pIgG_1_(κ) and pIgG_1_(λ) vectors were prepared by means of double digestion with *Dra*III/*Nhe*I and *Bsp*EI/*Nhe*I, respectively, and purified by means of agarose gel electrophoresis and gel extraction (gel purification kit, Qiagen). The assembly of antibody expression vector was carried out by means of homologous recombination with a Geneart Seamless Assembly Kit (Invitrogen) according to vendor's instructions as shown in Fig E1.

Briefly, 20 ng of paired VH and VL DNA segments, 50 ng of backbone insert, and 100 ng of linear vector were combined with Seamless enzyme mix and incubated at room temperature for 30 minutes. The assembly products were immediately transformed into competent *E coli* (One Shot Top 10, Invitrogen), and transformants were analyzed for the presence of DNA inserts by using colony PCR. The plasmid DNA of positive clones was purified and sequenced to ensure no base mutations and correct recombination.

Human embryonic kidney 293T cells (ATCC) were seeded in 6-well plates to produce cloned antibodies on a small scale for validating their binding specificities. Transient transfections were performed at 80% cell confluency by linear polyethylenimine with an average molecular weight of 25 kDa (Polysciences, Warrington, Pa) as a transfection reagent. The cells were cultured for 2 days before the supernatants were harvested and analyzed by means of ELISA for SAE binding reactivity. The concentrations of recombinant IgG_1_ antibodies released into the supernatants of transfected 293T cells were measured by means of ELISA with human IgG Quantitation Kits (Bethyl Laboratories, Montgomery, Tex). The γ1 constant regions of SAE-specific antibody expression vectors were replaced by the ε constant region or the C_H_1 domain of the γ1 constant region, to construct IgE and Fab expression vectors, respectively.

### ELISA for SAE-specific antibodies in culture supernatants

Microtiter plates were coated with 2 μg/mL of each SEA, SEB, SEC1, SED, SEE, and TSST-1 (Toxin Technology) or ovalbumin (50 μL/well) overnight at 4°C and blocked with 200 μL of assay diluent (0.5% BSA, 0.05% Tween-20, and 0.01% thimerosal in PBS). Bound IgG_1_ antibodies were detected by using horseradish peroxidase–conjugated goat anti-human IgG Fc antibody (Jackson ImmunoResearch, West Grove, Pa) and developed with NeA-Blue TMB substrate (Clinical Science Products, Mansfield, Mass).

### Basophil degranulation assay

RBL SX-38 cells were typically grown in complete MEM medium, as previously described.^E3^ One day before a planned experiment, RBL SX-38 cells were seeded at 6 × 10^5^ cells/mL (500 μL/well) in 48-well plates. After an overnight incubation, 250 μL of complete MEM medium containing SAE-specific IgE at 1 μg/mL was added to sensitize the cells. After a 2-hour incubation at 37°C, each well was washed twice with 500 μL of prewarmed Tyrode buffer (135 mmol/L NaCl, 5 mmol/L KCl, 5.6 mmol/L glucose, 1.8 mmol/L CaCl_2_, 1 mmol/L MgCl_2_, 20 mmol/L HEPES, and 0.5 mg/mL BSA, pH 7.3) and then 250 μL of Tyrode buffer containing SAEs at 1 μg/mL or polyclonal anti-human IgE antibody (Bethyl Laboratories) at 3 μg/mL or 1% Triton X-100 was added to the cells. After a 30-minute incubation in a 37°C incubator, culture media were collected and subjected to centrifugation at 300*g* for 5 minutes at room temperature. Fifty microliters of cleared supernatants were transferred to each well of a 96-well black OptiPlate (PerkinElmer, Waltham, Mass), and then 50 μL of substrate solution (80 μmol/L 4-methylumbelliferyl-N-acetyl-D-glucosaminide [Sigma-Aldrich] in 0.1 mol/L citric acid buffer, pH 4.5) was added to each well. The tape-sealed plate was incubated at 37°C for 1 hour, and the reaction was terminated by adding 100 μL of glycine buffer (0.2 mol/L glycine and 0.2 mol/L NaCl, pH 10.7). The resulting fluorescence (excitation, 355 nm; emission, 460 nm) was measured with a Victor 3 fluorescence reader (PerkinElmer). Measured values were expressed as percentages relative to the value of release (100%) obtained by lysing cells with 1% Triton X-100.

### SPR

Purified and concentrated immunoglobulin solutions were quantified with a NanoDrop spectrophotometer (Thermo Scientific, Wilmington, Del) by using absorbance at 280 nm, and the protein concentration was calculated with the extinction coefficient of each antibody, which was predicted by conducting the computation with a ProtParam program (http://web.expasy.org/protparam/). Biotin-labeled SEE and SEA were immobilized on a streptavidin-coated sensor chip (GE Healthcare). Binding of SAE-specific IgG_1_ antibodies was measured at 25°C by using a 3-minute association phase and a 10-minute dissociation phase (unless otherwise specified in the figure legend) in a concentration series ranging from 250 to 16 nmol/L. One antibody was injected for 3 minutes, followed by a 3-minute injection of the second antibody or buffer, to detect simultaneous binding of 2 antibodies. After regeneration with glycine-HCl (pH 2.5), the experiment was performed in reverse. Standard double-referencing data subtraction methods were used with a control surface (biotin alone).
